# Identification of common genes associated with diabetic nephropathy and diabetic retinopathy

**DOI:** 10.3389/fcell.2026.1808941

**Published:** 2026-04-28

**Authors:** Juan He, Dandan Zhang, Yan Wang

**Affiliations:** 1 Department of Nephrology, Xijing Hospital, Fourth Military Medical University, Xi’an, Shaanxi, China; 2 Department of Endocrinology, Honghui Hospital, Xi’an Jiaotong University, Xi’an, Shaanxi, China

**Keywords:** chronic kidney disease, diabetic nephropathy, diabetic retinopathy, diagnostic genes, regulate

## Abstract

**Background:**

There is a common basis of the diabetic nephropathy (DN) and diabetic retinopathy (DR), but the common genes of DN and DR were unclear.

**Methods:**

In this study, DN-related and DR-related transcriptome data GSE185011 was extracted. Firstly, the differentially expressed genes 1 (DEGs1) between DN and normal control (HC) samples, and DEGs2 between DR and HC samples were screened by “limma”. Then, the common genes of DN and DR were obtained by intersecting the DEGs1 and DEGs2. Next, the enrichment analyses of DEGs1 and DEGs2 were conducted by “ClusterProfiler”, and the enrichment analysis of common genes was conducted by “Metascape”. Moreover, the diagnostic genes were screened out by least absolute shrinkage and selection operator (LASSO) analysis, and the gene set enrichment analysis (GSEA) of diagnostic genes was performed. Furthermore, the immune cell infiltration analysis was used to further study the differences of disease mechanisms. In addition, the TF-mRNA-miRNA regulatory networks were constructed to reveal the potential regulation of diagnostic genes at the molecular level.

**Results:**

A totals of 206 common genes were obtained by intersecting the 526 DEGs1 and 1,059 DEGs2. Then, 6 diagnostic genes (METTL27, NMNAT2, TTC25, GAS6, ATP4A and ZNF223) of DN and 4 diagnostic genes (MCOLN3, C17orf77, ENPP3 and ATP4A) of DR were screened out. Notably, the proportion of central memeory CD4 T cells was significantly decreased in DN groups, and activated B cells was significantly increased in DR groups. Furthermore, the 493 TF-6mRNA-183miRNA regulatory network of DN and 111 TF-4mRNA-33miRNA regulatory network of DR were constructed, among them, hsa-mir-101-3p was the common miRNA for ZNF223, NMNAT2 and TTC25, and EGR1 was the key TF which could regulate ATP4A, GAS6, METTL27, NMNAT2 and TTC25 at the same time.

**Conclusion:**

This study revealed the potential molecular mechanisms of diagnostic genes in DN and DR, which could provide novel insights for the clinical diagnosis and treatment of DN and DR.

## 1 Introduction

Diabetes mellitus (DM) has become a global public health crisis, with its prevalence escalating rapidly over the past few decades. It is estimated that the global prevalence of diabetes will reach 783 million by 2045, imposing an enormous burden on healthcare systems worldwide (Washietl et al., 2004). Diabetic nephropathy (DN) and diabetic retinopathy (DR) are two major microvascular complications of DM, which are the leading causes of end-stage renal disease (ESRD) and irreversible blindness in working-age populations, respectively (Sarrigeorgiou et al., 2022). These complications not only severely compromise patients’ quality of life but also lead to substantial socioeconomic costs (Li, 2022). Both DN and DR share common risk factors such as prolonged hyperglycemia, hypertension, dyslipidemia, and obesity (Singh et al., 2018) Hyperglycemia-induced oxidative stress, endothelial dysfunction, and chronic inflammatory responses are widely recognized as core pathological processes underlying the development and progression of both complications (Kayid, 2021). For instance, high glucose levels can trigger the overproduction of reactive oxygen species (ROS), which disrupt the redox balance and damage vascular endothelial cells, a key initiating event in both renal and retinal microangiopathy (Cortés-Vázquez et al., 2020). Despite extensive research, the shared molecular mechanisms and specific regulatory networks between DN and DR remain not fully elucidated (Hegarty et al., 2022). Accumulating evidence suggests that there are common genetic and epigenetic regulatory patterns in the two complications, which may serve as potential targets for early diagnosis and targeted therapy (Hettmann et al., 2020). For example, microalbuminuria, an early marker of DN, has also been shown to be associated with an increased risk of DR, indicating a potential link in their pathological progression (Washietl et al., 2004). Additionally, recent studies have identified several common biomarkers, such as ATP4A, that may facilitate the simultaneous diagnosis of both DN and DR (Kang and Cho, 2023).

DN and DR share a complex pathophysiological foundation that extends across multiple molecular and cellular levels. The neurotrophin signaling pathway and chemokine signaling pathway have been implicated in the regulation of microvascular integrity in both the kidney and retina (Dahlgren et al., 2021). Dysregulation of these pathways may contribute to microvascular damage in DN and DR through distinct but overlapping mechanisms (McDermid et al., 2022). Furthermore, at the immune microenvironment level, immune cell dysregulation—including alterations in central memory CD4^+^ T cells and activated B cells—plays a critical role in the persistent inflammatory damage seen in both complications (Blakeney et al., 2021). Notably, the comorbid basis of DN and DR manifests across multiple mechanistic layers. The neurotrophin signaling pathway and chemokine signaling pathway have been implicated in the regulation of microvascular integrity in both the kidney and retina (Dahlgren et al., 2021). Dysregulation of these pathways may contribute to microvascular damage in DN and DR through distinct but overlapping mechanisms (McDermid et al., 2022). At the immune microenvironment level, immune cell dysregulation-including alterations in central memory CD4^+^ T cells and activated B cells-plays a critical role in the persistent inflammatory damage seen in both complications. In the context of trace element metabolism, zinc homeostasis disorders have also attracted increasing attention due to their involvement in the progression of both DN and DR. Impaired zinc transport in renal glomerular endothelial cells and abnormal zinc levels in retinal pigment epithelial cells have been shown to promote pathological changes in respective organs (Love et al., 2014). At the post-transcriptional regulatory level, non-coding RNAs, such as hsa-mir-101-3p and hsa-mir-124-3p, have been reported to be dysregulated in both complications, regulating endothelial cell apoptosis, oxidative stress, and retinal angiogenesis (Shannon et al., 2003). These shared pathological features across multiple layers suggest that DN and DR may be governed by an intertwined gene regulatory network. Given the high incidence and severe consequences of DN and DR, elucidating their common pathogenic mechanisms and potential biomarkers is critical for improving early diagnosis and developing effective therapeutic strategies. Given the high incidence and severe consequences of DN and DR, identifying their shared pathogenesis and potential biomarkers is crucial for improving early diagnosis and developing effective therapeutic strategies (Rigden and Fernández, 2021). Against this background, the present study aimed to systematically explore the common molecular basis and regulatory networks between DN and DR using comprehensive bioinformatics approaches. Based on public transcriptomic datasets, we performed differential expression analysis, functional enrichment analysis, and protein–protein interaction (PPI) network construction, and screened key genes via the least absolute shrinkage and selection operator (LASSO) regression diagnostic model. We further conducted immune cell infiltration analysis and constructed TF-mRNA-miRNA regulatory networks to elucidate the upstream regulatory mechanisms of key genes and their interaction with the immune microenvironment. This study aims to provide novel clues for understanding the comorbid mechanisms of DN and DR, and to lay a theoretical foundation for exploring potential biomarkers and therapeutic targets for their early diagnosis and targeted treatment.

## 2 Materials and methods

### 2.1 Data extraction

**FIGURE 1:**
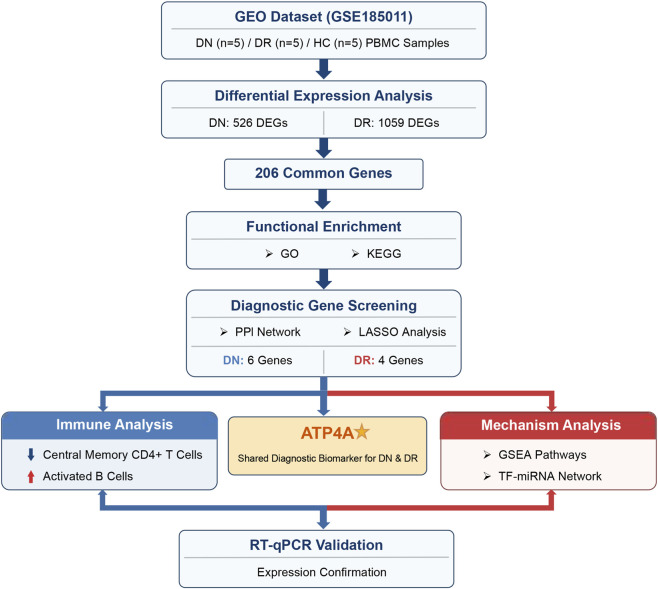
Workflow of the bioinformatics analysis for identifying shared biomarkers in DN and DR.

DN-related and DR-related transcriptome data were extracted from gene expression omnibus (GEO) database (https://www.ncbi.nlm.nih.gov/geo/). GSE185011 includes 5 DN, 5 DR and five normal control (HC) peripheral blood mononuclear cell samples. The overall bioinformatics analysis workflow of this study is shown in Figure 1.

DEGs were identified from GEO datasets, and common genes were obtained. Functional enrichment, PPI, and LASSO analyses were performed to screen diagnostic genes and construct models. ATP4A was identified as a shared biomarker. Subsequently, GSEA, immune infiltration, and regulatory network analyses were conducted to explore potential mechanisms, and RT-qPCR was used for validation.

### 2.2 Function enrichment analysis of common differentially expressed genes (common genes) of DN and DR

Firstly, the differentially expressed genes 1 (DEGs1) between 5 DN and 5 HC samples and DEGs2 between 5 DR and 5 HC samples were compared by “DESeq2” R package (version 3.44.3) (|log_2_ fold change (FC)| > 1, *p* < 0.05), respectively (Love et al., 2014). The function enrichment analyses of DEGs1 and DEGs2 were conducted by “clusterprofiler” R package (version 3.16.0) with an adjusted p-value (adj.p.value <0.05) as the significance threshold (PMID: 34557778). Then, the common genes with the consistent expression trends of DEGs1 and DEGs2 were obtained using “venn” (http://bioinformatics.psb.ugent.be/webtools/Venn/). Besides, the function enrichment analyses of these common genes were conducted by “Metascape” (http://metascape.org/gp/index.html#/main/step1). To further visualize the functional relationships between common genes and representative biological processes, gene–pathway interaction subnetworks were constructed using Cytoscape software (version 3.9.1)^15^.

### 2.3 Screening of the diagnostic genes

In this study, the protein-protein interaction (PPI) network was constructed to investigate the relationships among common genes by the “STRING”, and the hub genes were obtained with confidence equal to 0.4 (https://string-db.org) (Rigden and Fernández, 2021). Then, the diagnostic genes were screened by least absolute shrinkage and selection operator (LASSO) analysis, and the diagnostic models of DN and DR were constructed, respectively. To prevent overfitting, internal cross-validation was applied during model construction, and the optimal penalty parameter (λ) was determined accordingly. Besides, the receiver operating characteristic (ROC) curves were drawn to assess the diagnose ability of the 2 diagnostic models and their diagnostic genes. In addition, the expression levels and correlations among these diagnostic genes were calculated by “psych” R package (version 2.0.9).

### 2.4 The gene set enrichment analysis (GSEA) of diagnostic genes

The correlation coefficient between each diagnostic gene and all genes were calculated, and GSEA of all diagnostic genes were performed by “clusterProfiler” R package (|NES| > 1, adj. *p*.value <0.05) (Yu et al., 2012).

### 2.5 Analysis of immune micro-environment

In this study, the proportions of 28 immune cells were obtained by “ssGSEA” algorithm, and the differences between DN and HC, DR and HC were compared by “Wilcoxon” analysis, respectively. Then, the correlations between diagnostic genes and the differentially immune cells were calculated by “Spearman” analysis.

### 2.6 Construction of TF-mRNA-miRNA regulatory networks

Firstly, the targeted transcription factors (TFs) were screened out by “ChEA3” online tool (https://amp.pharm.mssm.edu/chea3/) and the targeted miRNAs were predicted by miRNet (http://www.mirnet.ca/). Then, the TF-mRNA-miRNA networks of 2 groups of diagnostic genes were constructed by “Cytoscape”, respectively (version 3.8.2)^15^.

### 2.7 Expression verification of diagnostic genes

The reverse transcription quantitative polymerase chain reaction (RT-qPCR) was performed to validate the expression of diagnostic genes. These samples were committed by the patients and the Ethics Committee of Ethics Committee of Xi’an First Hospital (2017 (Ethical Review No. 5)). Total RNA was extracted using TRIZol (Thermo Fisher, Shanghai, CN), and mRNA was reverse transcribed into cDNA and RT-qPCR reactions using SureScript-First-strand-cDNA-synthesis-kit (Servicebio, WuHan, CN). Primers were shown in Table S1 in the Supplementary Materials.

## 3 Results

### 3.1 Totals of 206 common genes were associated with both DN and DR

**FIGURE 2:**
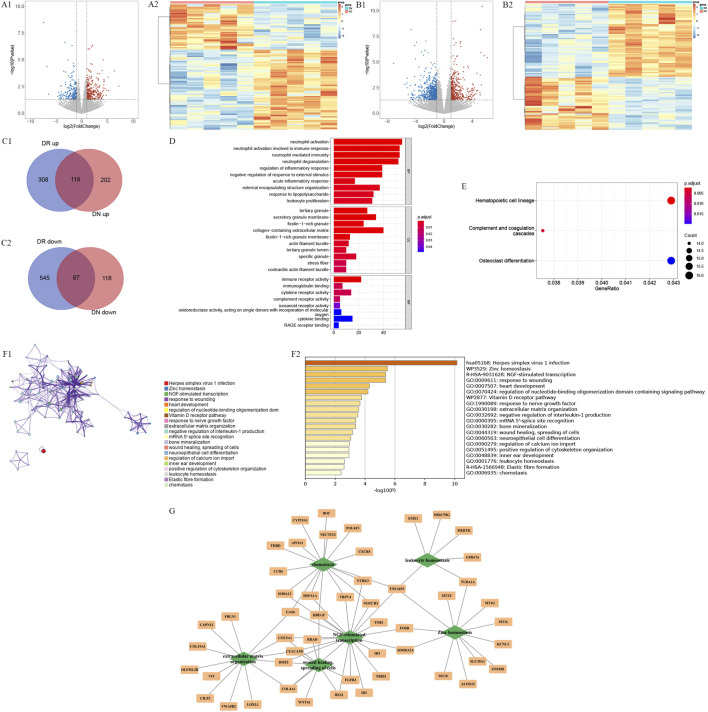
Identification of differentially expressed genes (DEGs) and common genes between diabetic nephropathy (DN) and diabetic retinopathy (DR), and functional enrichment analysis of these genes. **(A,B)** Volcano plots of differentially expressed genes (DEGs) between DN vs. healthy control (HC) **(A)** and DR vs. HC **(B)** groups. **(C)** Venn diagram showing the overlap of DEGs between DN and DR. **(D,E)** Functional enrichment analysis of DEGs in DR. **(D)** Bubble plot of Gene Ontology (GO) biological process enrichment analysis. **(E)** Bubble plot of Kyoto Encyclopedia of Genes and Genomes (KEGG) pathway enrichment analysis. **(F)** Functional enrichment analysis of the common genes between DN and DR using Metascape. **(G)** Gene-pathway interaction networks constructed for the common DEGs between DN and DR, showing representative enriched pathways and their associated genes.

There were 526 DEGs1 (321 upregulated and 205 downregulated) between 5 DN and 5 HC samples, and 1,059 DEGa2 (427 upregulated and 632 downregulated) between 5 DR and 5 HC samples (Figures 2A,B; Supplementary Tables S2, S3). Then, 206 common genes (119 upregulated and 87 downregulated) were obtained (Figure 2C; Supplementary Table S4).

Besides, the DEGs1 were not enriched to Gene Ontology (GO) function and Kyoto Encyclopedia of Genes and Genomes (KEGG) pathway, the DEGs2 were enriched to 64 GO functions, including neutrophil activation involved in immune response, negative regulation of response to external stimulus and etc., In addition, these genes were enriched to 3 KEGG pathways, including hematopoietic cell lineage, complement and coagulation cascades and osteoclast differentiation (Figures 2D,E). It was worth noting that these 206 common genes were enriched to 140 terms, including herpes simplex virus 1 infection, zinc homeostasis, NGF-stimulated transcription and etc. (Figure 2F). To provide a more intuitive understanding of the functional roles of these common genes, representative enriched pathways were selected from the top enriched terms based on enrichment significance and disease relevance, and gene–pathway interaction subnetworks were constructed (Figure 2G). As shown in the network, pathways such as leukocyte homeostasis, chemotaxis, extracellular matrix organization, wound healing, NGF-stimulated transcription, and zinc homeostasis were linked to multiple common genes. For example, CCR6 and CXCR5 were involved in chemotaxis, COL4A1 and COL5A1 were enriched in extracellular matrix organization, and MT1X and SLC30A1 were associated with zinc homeostasis. These subnetworks suggested that the common genes may participate in immune regulation, tissue remodeling, and ion homeostasis–related processes.

### 3.2 6 diagnostic genes of DN and 4 diagnostic genes of DR were screened

**FIGURE 3:**
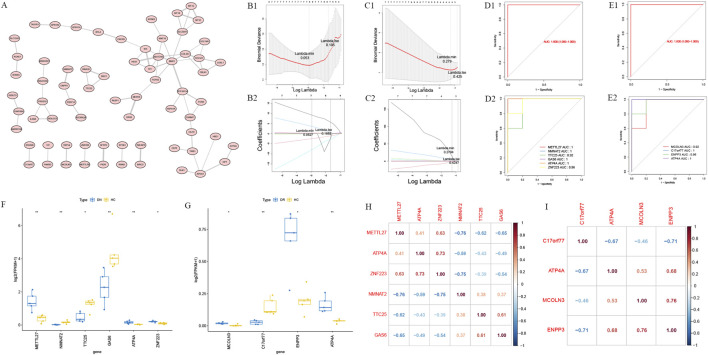
Screening and characterization of diagnostic genes for diabetic nephropathy (DN) and diabetic retinopathy (DR). **(A)** Protein-protein interaction (PPI) network diagram based on the 206 common genes. **(B1,B2)** LASSO regression coefficient path diagrams of diagnostic genes for DN and DR. **(C1,C2)** Cross-validation curve diagrams of LASSO regression models for DN and DR. **(D1,D2)** Receiver Operating Characteristic (ROC) curve diagrams of diagnostic models for DN and DR. **(E1,E2)** ROC curve diagrams of each diagnostic gene for DN and DR. **(F)** Box plots of expression levels of DN diagnostic genes in healthy control (HC), DN, and DR groups. **(G)** Box plots of expression levels of DR diagnostic genes in healthy control (HC), DN, and DR groups. **(H)** Correlation heatmap of DN diagnostic genes. **(I)** Correlation heatmap of DR diagnostic genes.

In this study, the PPI network of 206 common genes was constructed and 69 hub genes were obtained (Figure 3A; Supplementary Table S5). Then, 6 diagnostic genes (METTL27, NMNAT2, TTC25, GAS6, ATP4A and ZNF223) of DN and 4 diagnostic genes (MCOLN3, C17orf77, ENPP3 and ATP4A) of DR were screened by LASSO, respectively (Figures 3B,C).

Based on it, the diagnostic models of DN and DR were constructed, respectively. The areas under ROC curve (AUC value) of these 2 model were 1, and the AUC value of all these diagnostic genes were great than 0.9, all these results showed that these 2 models could be used as effective diagnostic models (Figures 3D,E). For ATP4A, the optimal cut-off values were determined, along with the corresponding sensitivity and specificity. In the DR group, the optimal cut-off value was 0.306, with a sensitivity of 1 and a specificity of 1. In the DN group, the optimal cut-off value was 0.212, with a sensitivity of 1 and a specificity of 1. In addition, ATP4A showed high diagnostic performance in both DN and DR groups, with AUC values greater than 0.9. Moreover, the expression levels of these diagnostic genes were compared, among them, the expression of NMNAT2, TTC25 and GAS6 was decreased significantly, and that of METTL27, ATP4A and ZNF223 were increased significantly in DN groups, the expression of C17orf77 was decreased significantly, and MCOLN3, ENPP3 and ATP4A were increased significantly in DR groups (Figures 3F,G). Besides, ZNF223 was positively associated with ATP4A (cor = 0.73) and negatively associated with NMNAT2 (cor = −0.75) significantly, METTL27 was negatively associated with NMNAT2 (cor = −0.76) significantly. On the other hand, ENPP3 was positively associated with MCOLN3 (cor = 0.76) and negatively associated with C17orf77 (cor = −0.71) significantly (Figures 3H,I).

### 3.3 The GSEA of diagnostic genes

**FIGURE 4:**
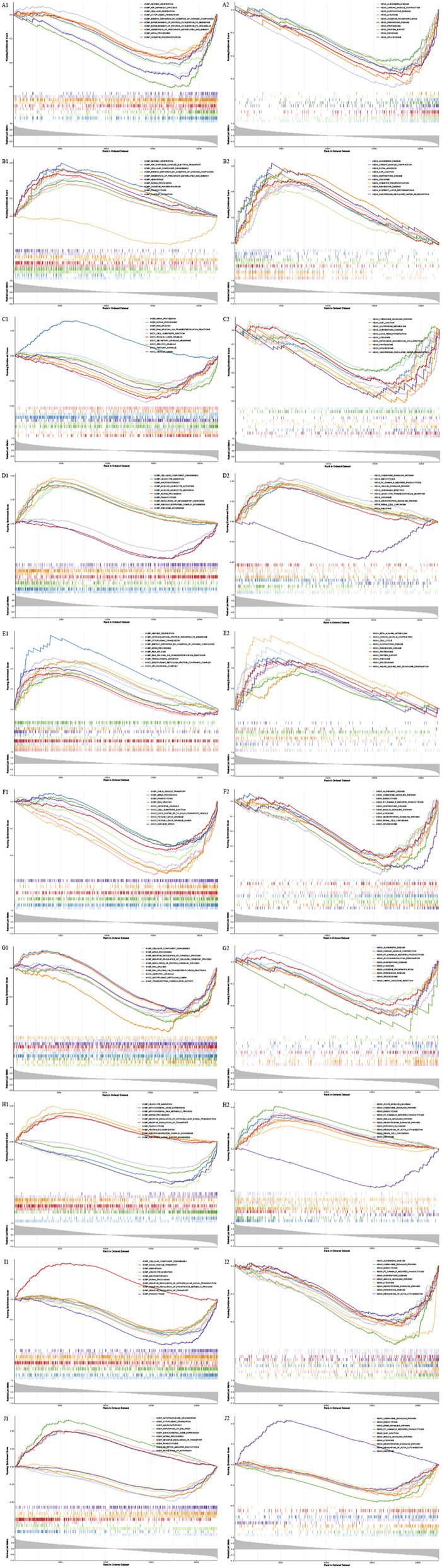
Gene Set Enrichment Analysis (GSEA) of diagnostic genes for diabetic nephropathy (DN) and DR). Gene Set Enrichment Analysis (GSEA) of diagnostic genes for diabetic nephropathy (DN) and diabetic retinopathy (DR). **(A–F)** Gene Set Enrichment Analysis (GSEA) result diagrams of DN diagnostic genes METTL27, NMNAT2, TTC25, GAS6, ATP4A, and ZNF223, respectively. **(G–J)** Gene Set Enrichment Analysis (GSEA) result diagrams of DR diagnostic genes MCOLN3, C17orf77, ENPP3, and ATP4A, respectively. **(A)** Protein-protein interaction (PPI) network diagram based on the 206 common genes. **(B1,B2)** LASSO regression coefficient path diagrams of diagnostic genes for DN and DR. For each panel: The top curve represents the enrichment score (ES) of the gene set; the middle heatmap shows the expression of genes in the set; the bottom bar plot indicates the position of genes in the ranked list (red = upregulated, blue = downregulated).

The results of GSEA of 6 diagnostic genes of DN were shown in the Figures 4A–F. The results revealed that most of the diagnostic genes of DN were associated with the biological process of mRNA processing. Noticeable, the functions of ATP4A and TTC25 were similar, which were associated with aerobic respiration, cytoplasmic translation, energy derivation by oxidation of organic compounds, and etc., and GAS6 played opposite roles in these processes. The functions of METTL27 and TTC25 were associated with RNA splicing via transesterification reactions, and the functions of METTL27 and ZNF223 were associated with RNA splicing, cell substrate junction, ficolin 1 rich granule, and etc. On the other hand, most of diagnostic genes were associated with the pathway of huntingtons disease and lysosome. Noticeable, the pathways of NMNAT2 and ZNF223 were similar, which were associated with endocytosis, FC gamma R mediated phagocytosis, insulin signaling pathway, neurotrophin signaling pathway, and etc., The pathways of ATP4A, METTL27, TTC25 and ZNF223 were associated with spliceosome, ATP4A, GAS6 and TTC25 were associated with cardiac muscle contraction, parkinsons disease, ATP4A, GAS6 and ZNF223 were associated with alzheimers disease.

The results of GSEA of 4 diagnostic genes of DR were shown in the Figures 4G–J. The results revealed that C17orf77, ENPP3 and MCOLN3 were associated with biological process of ncRNA processing, negative regulation of transport, phagocytosis and etc., Noticeable, the function of cellular component disassembly was negative with ENPP3, and positive with ATP4A. On the other hand, the pathway of FC gamma R mediated phagocytosis was negative with ATP4A and ENPP3, and positive with C17orf77 and MCOLN3. Besides, the pathway of lysosome was highly enriched in ATP4A, ENPP3 and MCOLN3, the pathways of chemokine signaling pathway, endocytosis, insulin signaling pathway, neurotrophin signaling pathway, regulation of actin cytoskeleton were highly enriched in C17orf77, ENPP3 and MCOLN3.

### 3.4 Immune cell infiltration patterns in DN and DR

**FIGURE 5:**
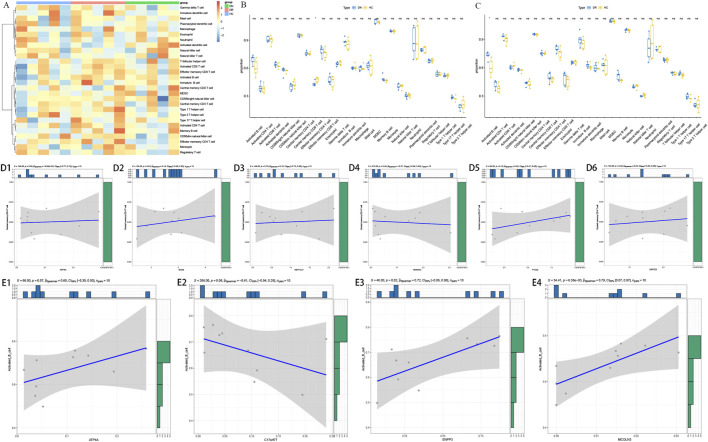
Immune cell infiltration profiles and their correlation with diagnostic genes in diabetic nephropathy (DN), diabetic retinopathy (DR), and healthy control (HC) groups. **(A)** Heatmap of the relative proportions of 28 types of immune cells in healthy control (HC), diabetic nephropathy (DN), and diabetic retinopathy (DR) groups. **(B)** Violin plot comparing the proportion of central memory CD4^+^ T cells among different groups. **(C)** Violin plot comparing the proportion of activated B cells among different groups. **(D1–D6)** Correlation scatter plots between the proportion of central memory CD4^+^ T cells and the expression level of each DN diagnostic gene. **(E1–E4)** Correlation scatter plots between the proportion of activated B cells and the expression level of each DR diagnostic gene.

The proportion of the 28 immune cells in DN, DR and HC groups were showed in Figure 5A. The proportion of central memory CD4 T cells was significantly decreased in DN groups, and activated B cell was significantly increased in DR groups (Figures 5B,C). Furthermore, the proportion of central memory CD4^+^ T cells was not significantly associated with any DN diagnostic genes (Figures 5D1–D6). In DR, activated B cells showed significant positive correlations with ENPP3 (ρ = 0.72, P = 0.02) and MCOLN3 (ρ = 0.79, P = 6.38 × 10^−3^) (Figures 5E1,E2). In addition, ATP4A showed a positive correlation trend, while C17orf77 showed a negative correlation trend, neither of which reached statistical significance (Figures 5E3,E4).

### 3.5 Molecular mechanism analysis

**FIGURE 6:**
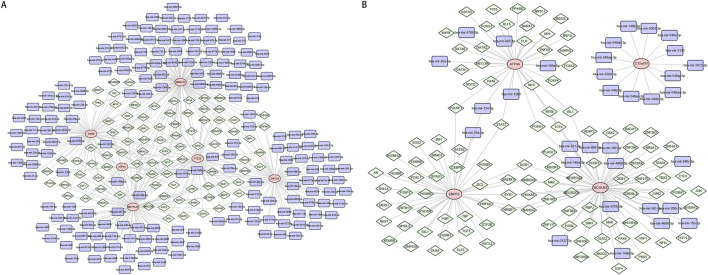
TF-mRNA-miRNA regulatory networks of diagnostic genes for diabetic nephropathy (DN) and diabetic retinopathy (DR). **(A)** TF-mRNA-miRNA regulatory network diagram of diabetic nephropathy (DN) diagnostic genes. **(B)** TF-mRNA-miRNA regulatory network diagram of diabetic retinopathy (DR) diagnostic genes.

A total of 493 TFs and 183 miRNAs associated with diagnostic genes of DN were downloaded and 400 relationship pairs were obtained for constructing the TF-mRNA-miRNA regulatory network of DN (Figure 6A; Supplementary Table S6). In this network, hsa-mir-101-3p was the common miRNA for ZNF223, NMNAT2 and TTC25, hsa-mir-124-3p and hsa-mir-146a-5p were the common miRNAs of GAS6 and ATP4A, hsa-mir-190a-5p and hsa-mir-634 were the common miRNAs of ZNF223 and GAS6, hsa-mir-195-5p and hsa-mir-128-3p were the common miRNAs of NMNAT2 and GAS6, hsa-mir-210-3p and hsa-mir-1343-3p were the common miRNAs of NMNAT2 and ZNF223. EGR1 was the key TF which could regulate ATP4A, GAS6, METTL27, NMNAT2 and TTC25 at the same time, there were five common TFs (BHLHE22, VDR, REST, TP53, ESR1) of GAS6, METTL27 and NMNAT2, 4 (HNF4A, ELK1, SETDB1, MAFF) common TFs of METTL27, TTC25 and ZNF223, 3 common TFs (JUND, JUN, GATA4) of GAS6, METTL27 and TTC25, 3 common TFs (POU5F1, KLF5, NR3C1) of ATP4A, METTL27 and NMNAT2.

A total of 111 TFs and 33 miRNAs associated with diagnostic genes of DR were downloaded and 158 relationship pairs were obtained for constructing the TF-mRNA-miRNA regulatory network of DR (Figure 6B; Supplementary Table S7). In this network, we found that hsa-mir-124-3p was the common miRNA of ATP4A and ENPP3. There were 7 common TFs (SREBF1, EGR1,NANOG, NR0B1, HMBOX1, EVX1, HOXA5) of ENPP3 and MCOLN3, 4 common TFs (RFX6, ISL1, FOXA1, TCF4) of ATP4A and MCOLN3, 2 common TFs (POU5F1, STAT3) of ATP4A and ENPP3.

### 3.6 Expression verification of diagnostic genes

**FIGURE 7:**
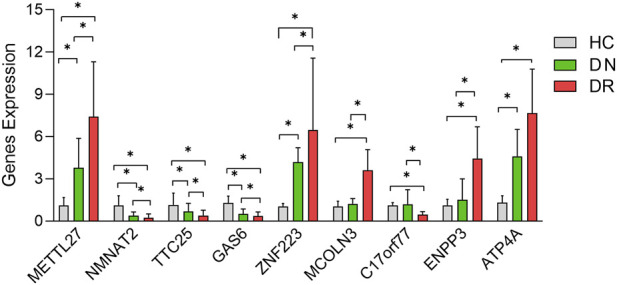
Validation of diagnostic gene expression levels by qRT-PCR. The bar graphs show the relative expression levels of 6 DN diagnostic genes (METTL27, NMNAT2, TTC25, GAS6, ATP4A, ZNF223) and 4 DR diagnostic genes (MCOLN3, C17orf77, ENPP3, ATP4A) in HC, DN and DR groups. The y-axis represents the relative mRNA expression normalized to the internal reference gene, and the x-axis represents the different gene groups. Data are presented as mean ± SD. *p < 0.05, **p < 0.01 vs. HC group.

The RT-qPCR results of 6 diagnostic genes of DN and 4 diagnostic genes of DR were showed that the expression trends of these genes were consistent with the transcriptome data analysis results. Specifically, in DN samples, the expression levels of METTL27, ATP4A and ZNF223 were significantly upregulated, while the expression levels of NMNAT2, TTC25 and GAS6 were significantly downregulated compared with HC samples (p < 0.05). In DR samples, the expression levels of MCOLN3, ENPP3 and ATP4A were significantly upregulated, while the expression level of C17orf77 was significantly downregulated compared with HC samples (p < 0.05). These results verified the reliability of the transcriptome data and the accuracy of the screened diagnostic genes. (Figure 7).

## 4 Discussion

DN and DR are major microvascular complications of diabetes mellitus, which severely compromise patients’ quality of life and impose a heavy burden on global healthcare systems (Shuvalova et al., 2021). Despite extensive research on the pathogenesis of DN and DR, the shared molecular mechanisms underlying their development and progression remain not fully elucidated (Guan et al., 2023). Identifying common biomarkers and exploring their regulatory networks are crucial for achieving early diagnosis and developing targeted therapeutic strategies for both complications (Hettmann et al., 2020). In this study, through systematic analysis of transcriptomic data from DN and DR, 206 common differentially expressed genes (DEGs) were identified in both complications, and these genes were significantly enriched in pathways such as zinc homeostasis. Further, through protein-protein interaction (PPI) network and LASSO regression analysis, 6 diagnostic genes for DN (METTL27, NMNAT2, TTC25, GAS6, ATP4A, ZNF223) and 4 diagnostic genes for DR (MCOLN3, C17orf77, ENPP3, ATP4A) were screened out, among which ATP4A was shared by both. The diagnostic models constructed based on these diagnostic genes exhibited excellent diagnostic efficacy, indicating that these genes have high potential as diagnostic biomarkers. Gene Set Enrichment Analysis (GSEA) suggested that these diagnostic genes are closely associated with pathways such as neurotrophin signaling, spliceosome, and chemokine signaling, respectively. Subsequently, we further explored their regulatory mechanisms and associations with the immune microenvironment, which provides new insights into the common pathogenic mechanisms of these two diabetic complications. The key findings of this study highlight the existence of common pathological processes, regulatory networks, and potential biomarkers between DN and DR, which may lay the foundation for the development of integrated diagnostic and therapeutic strategies. Of note, no significant GO/KEGG enrichment was observed for DN-related differentially expressed genes (DEGs) under the adjusted p-value threshold. This may be attributed to several factors. First, the relatively small sample size may have reduced statistical power, making it difficult to detect significant enrichment following multiple testing correction. Second, the use of peripheral blood mononuclear cell samples instead of renal tissue specimens may not fully reflect the molecular alterations occurring in the target organ. Furthermore, compared with diabetic retinopathy (DR), the functional changes of DN-related DEGs may be relatively subtle. Therefore, the absence of significant enrichment should be interpreted with caution.

The identification of 206 common genes between DN and DR samples laid a foundation for exploring their shared pathogenic mechanisms. Functional enrichment analysis revealed that these common genes were significantly enriched in terms such as herpes simplex virus 1 infection, zinc homeostasis, and NGF-stimulated transcription. Among these, zinc homeostasis attracted particular attention, which is consistent with previous studies reporting the involvement of zinc metabolism disorders in the progression of both DN and DR. Zinc, as an essential trace element, participates in various biological processes including oxidative stress regulation, inflammatory response, and endothelial function maintenance. Dysregulation of zinc homeostasis can induce oxidative stress and endothelial dysfunction, which are well-recognized core pathological processes in diabetic microvascular complications (Kayid, 2021), consistent with our results confirming that oxidative stress and endothelial dysfunction are core shared pathological mechanisms of DN and DR. High glucose-induced ROS overproduction can damage vascular endothelial cells, leading to increased vascular permeability, which is an early pathological feature of both renal glomerular damage and retinal microangiopathy (Cortés-Vázquez et al., 2020). For instance, a previous study demonstrated that impaired zinc transport in renal glomerular endothelial cells contributes to mesangial matrix expansion in DN, while abnormal zinc levels in retinal pigment epithelial cells are associated with DR progression (Hegarty et al., 2022). Therefore, the enrichment of common genes in zinc homeostasis in our study further confirms that zinc metabolism disorder may be a key common link between DN and DR, providing a potential shared therapeutic target for both diseases.

An important finding of this study is the identification of ATPase H+/K+ exchange transporter alpha subunit (ATP4A) as a potential common biomarker for both DN and DR. Specifically, RT-qPCR experiments demonstrated that the expression level of ATP4A was significantly upregulated in both DN and DR groups compared with the control group. ATP4A encodes the catalytic alpha subunit of gastric H^+^/K^+^-ATPase (proton pump), which is primarily responsible for gastric acid secretion (Abe et al., 2012). Notably, it has also been reported to be expressed in extragastric tissues such as the kidney (Barnawi et al., 2020), laying a foundation for its potential role in diabetic microvascular complications. Currently, direct research on ATP4A in diabetic microvascular complications remains limited; however, its role in the context of autoimmunity and metabolic disorders has garnered increasing attention. Multiple studies have indicated that ATP4A can function as an autoantigen, inducing autoimmune responses in patients with type 1 diabetes, and its autoantibodies are closely associated with disease phenotypes, hematological parameters, and *Helicobacter pylori* infection status (Chobot et al., 2014; Chobot et al., 2018; Wenzlau et al., 2015). In addition to its involvement in autoimmunity, loss-of-function mutations in ATP4A have been linked to the development of gastric neuroendocrine tumors (Ihara et al., 2020). Relevant to DR, elevated levels of ATP metabolites in the vitreous humor of DR patients have been shown to indicate local disorders of energy and purine signaling (Loukovaara et al., 2015), indirectly suggesting a potential role of ATP4A in DR pathogenesis. Based on the above evidence, we speculate that the upregulation of ATP4A in both DN and DR may be attributed to its involvement in regulating local tissue pH/ion homeostasis, triggering autoimmune responses, or mediating adaptation to cellular metabolic stress. To the best of our knowledge, this study is the first to reveal the common upregulation of ATP4A in the peripheral blood of these two diabetic microvascular complications, providing new evidence for its potential as a shared diagnostic biomarker. Nevertheless, the specific pathological mechanisms by which ATP4A exerts its effects in target tissues (e.g., kidney and retina) require further in-depth investigation. For DR, the enrichment of diagnostic genes in the chemokine signaling pathway confirms the critical role of chemokine-mediated inflammatory cell infiltration in retinal microangiopathy (Dahlgren et al., 2021), which is consistent with previous studies indicating the same mechanism (Fukunaga and Rao, 2022). Additionally, the expression patterns of diagnostic genes were consistent with their functional roles: for example, NMNAT2, which is involved in neurotrophin signaling pathway, was downregulated in DN, and its decreased expression may impair renal microvascular protection, thereby promoting DN progression. Transcription factors such as SREBF1 and EGR1, which co-regulate ENPP3 and MCOLN3 in DR, are involved in lipid metabolism and inflammatory responses, further supporting the link between metabolic disorders and inflammation in DR pathogenesis (McDermid et al., 2022) and the reliability of our regulatory network.

The constructed TF-mRNA-miRNA regulatory networks further revealed the molecular mechanisms underlying the regulation of diagnostic genes in DN and DR. In the DN regulatory network, EGR1 was identified as a key TF that simultaneously regulates five diagnostic genes (ATP4A, GAS6, METTL27, NMNAT2, TTC25). EGR1 has been reported to be involved in the regulation of oxidative stress and inflammatory response in diabetic complications (Lorenzen et al., 2021); its overexpression can promote the expression of pro-inflammatory cytokines, thereby exacerbating microvascular damage. The regulation of multiple diagnostic genes by EGR1 suggests that it may act as a central hub in the co-pathogenesis of DN. Regarding miRNAs, hsa-mir-101-3p was a common miRNA targeting ZNF223, NMNAT2, and TTC25. Immune cell dysregulation is another important shared feature of DN and DR. Non-coding RNAs also play important regulatory roles in the shared pathogenesis of DN and DR. As mentioned earlier, hsa-mir-101-3p is downregulated in both complications, and its overexpression can inhibit endothelial cell apoptosis and oxidative stress (Cortés-Vázquez et al., 2020), which is consistent with previous studies (Liang et al., 2018). The targeting of multiple diagnostic genes by hsa-mir-101-3p in our network indicates that it may regulate the progression of DN by coordinating the expression of these genes. In the DR regulatory network, hsa-mir-124-3p acts as a common miRNA for ATP4A and ENPP3, regulating retinal angiogenesis (Fukunaga and Rao, 2022); its dysregulation may contribute to DR-related retinal microvascular proliferation, as reported in previous studies (Shaik, 2019). These findings suggest that non-coding RNAs may serve as potential therapeutic targets for both DN and DR.

Immune infiltration analysis in this study revealed a significant reduction in central memory CD4^+^ T cells in patients with diabetic nephropathy (DN), and a marked increase in activated B cells in those with diabetic retinopathy (DR). These findings are largely consistent with the notion that immune dysregulation contributes to both DN and DR, even though the dominant immune signatures may differ between the two complications. In diabetic kidney disease/diabetic nephropathy (DKD/DN), accumulating evidence highlights a crucial role of CD4^+^ T cell-mediated inflammation in renal injury, interstitial fibrosis, and declining renal function. For instance, recent clinicopathological data have shown that greater CD4^+^ T cell infiltration in DN patients is associated with more severe tubular atrophy/interstitial fibrosis and poorer renal outcomes (Liu et al., 2023; Han et al., 2024).

Notably, although central memory CD4^+^ T cells were decreased in DN patients in the present study, they showed no significant correlation with any of the DN diagnostic genes. This suggests that the reduction in central memory CD4^+^ T cells may reflect a broader state of immune dysregulation in DN, rather than a direct linear association with the expression of individual biomarkers. Given the limited sample size and the complexity of immune regulation in DN, the absence of significant gene-immune correlations should be interpreted with caution. In contrast, activated B cells were increased in DR and were significantly positively correlated with ENPP3 and MCOLN3, whereas ATP4A and C17orf77 showed non-significant correlative trends. This pattern may imply that the identified DR-related diagnostic genes are more closely linked to alterations in the immune microenvironment compared with the genes identified in DN (Liu et al., 2023; Liao et al., 2024).

From a mechanistic perspective, the role of adaptive immune activation in DR has gained increasing attention. Reviews on the immunopathology of DR emphasize that chronic retinal inflammation involves leukocyte activation, vascular leakage, and disruption of the blood-retinal barrier (Yue et al., 2022). Meanwhile, studies in human specimens have reported elevated levels of B cell-derived antibodies in the vitreous humor of DR patients, supporting the involvement of B cell-related immune responses in disease progression (Liu et al., 2020). Furthermore, studies on circulating immune cells have indicated that peripheral immune cells can directly impair retinal endothelial function and contribute to DR pathogenesis (Liao et al., 2024). Therefore, the positive correlations of activated B cells with ENPP3 and MCOLN3, as well as the trending correlation with ATP4A in this study, suggest that these biomarkers not only possess diagnostic value but also reflect immune activation status in DR.

Clinically, such gene-immune cell associations may help identify patients with a more pronounced inflammatory phenotype and provide insights for future immune-based stratification and therapeutic target selection. Nevertheless, the causal relationships underlying these associations warrant further validation in larger cohorts and functional experiments.

In summary, the present study identified 206 common genes between DN and DR, screened specific diagnostic genes for each complication, and constructed TF-mRNA-miRNA regulatory networks. Additionally, we revealed the differences in immune cell infiltration between DN and DR and their correlations with diagnostic genes, providing new biomarkers and potential therapeutic targets for the diagnosis and treatment of DN and DR. In conclusion, DN and DR share common pathological processes (oxidative stress, endothelial dysfunction, inflammation), regulatory networks (neurotrophin signaling pathway, chemokine signaling pathway), and potential biomarkers (ATP4A). The identification of these shared features provides new opportunities for the integrated management of DN and DR. Targeting the common pathways and biomarkers may lead to the development of novel therapeutic strategies that can simultaneously prevent or treat both complications, improving the prognosis of diabetes patients.

This study has several limitations. First, it is a bioinformatics analysis based on public database data and a descriptive analysis based on existing literature, and the results need to be verified by *in vitro* and *in vivo* experiments to validate the shared molecular mechanisms and potential biomarkers identified. Second, the sample size and heterogeneity of the included studies may affect the generalizability of the results. Future prospective cohort studies with large sample sizes are required to confirm the clinical value of the identified biomarkers and regulatory pathways. Moreover, the specific molecular mechanisms by which diagnostic genes regulate the immune microenvironment and participate in the progression of DN and DR deserve further investigation.

## Data Availability

All datasets analyzed in this study were obtained from the public Gene Expression Omnibus (GEO) database. The dataset GSE185011 is publicly available at https://www.ncbi.nlm.nih.gov/geo/query/acc.cgi?acc=GSE185011. All results generated in this study are included in the article and Supplementary Materials.
