# Supplementary figures and images for: Identification of common genes associated with diabetic nephropathy and diabetic retinopathy
Source: Front Cell Dev Biol. 2026 Apr 28;14:1808941. doi: 10.3389/fcell.2026.1808941 (PMC13160884; doi:10.3389/fcell.2026.1808941)

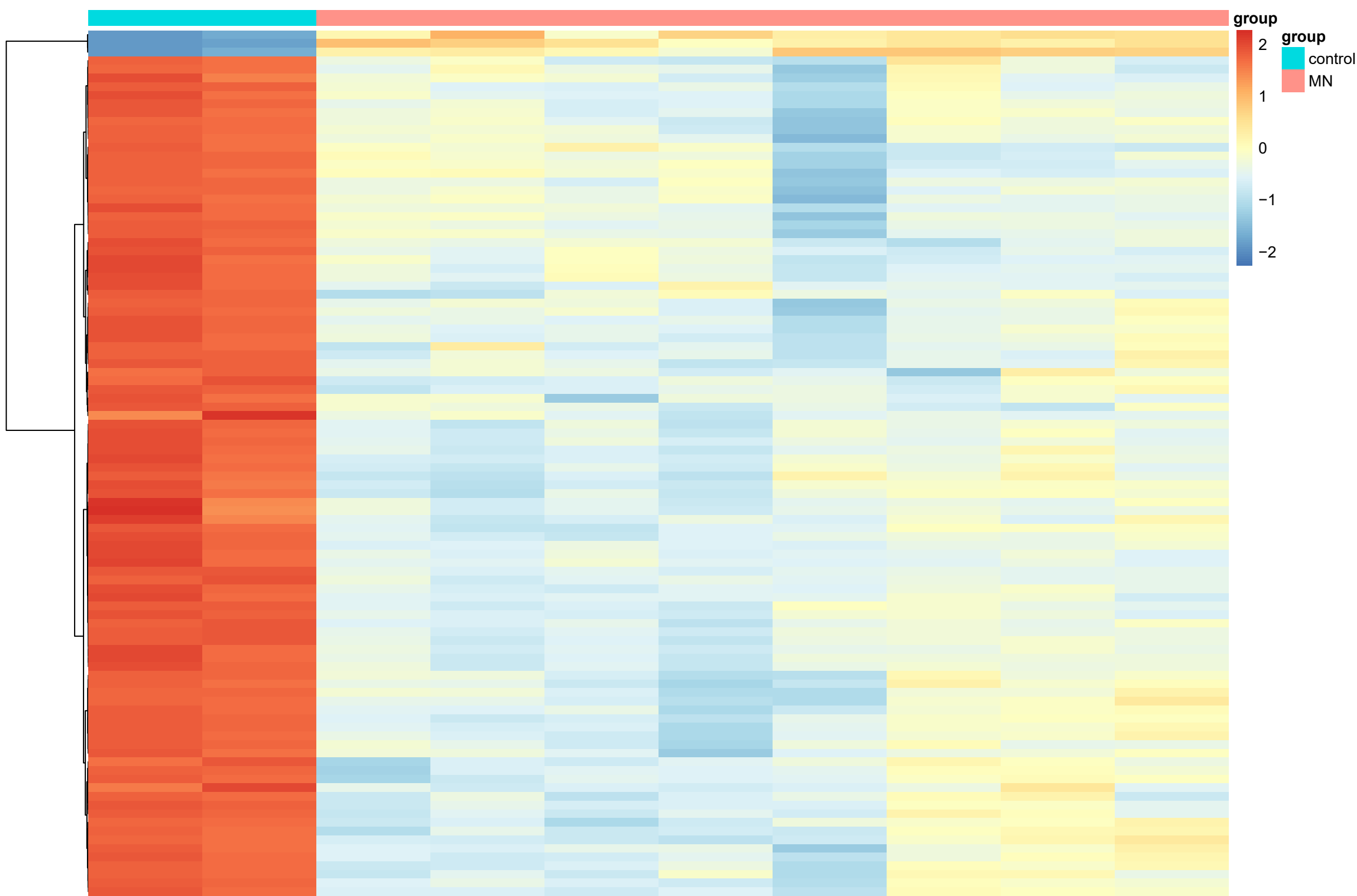

Supplement: Supplementary file 1 [file DataSheet2.pdf]

DR down

DN down

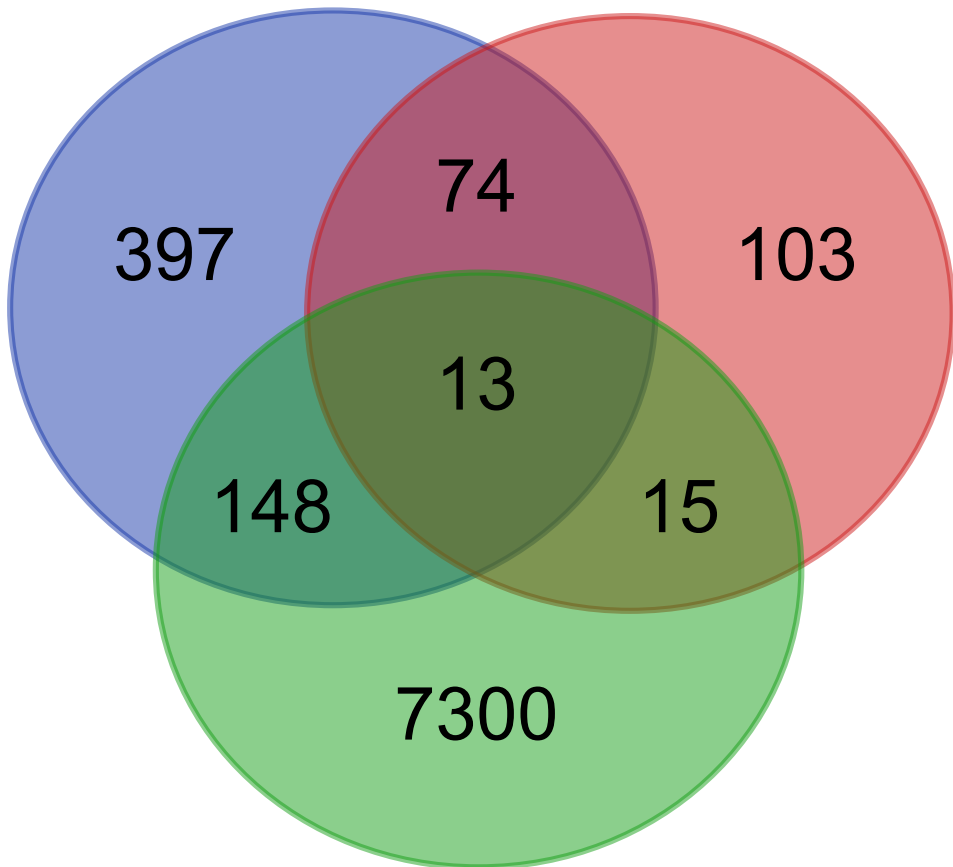

MN down

Supplement: Supplementary file 3 [file DataSheet4.pdf]

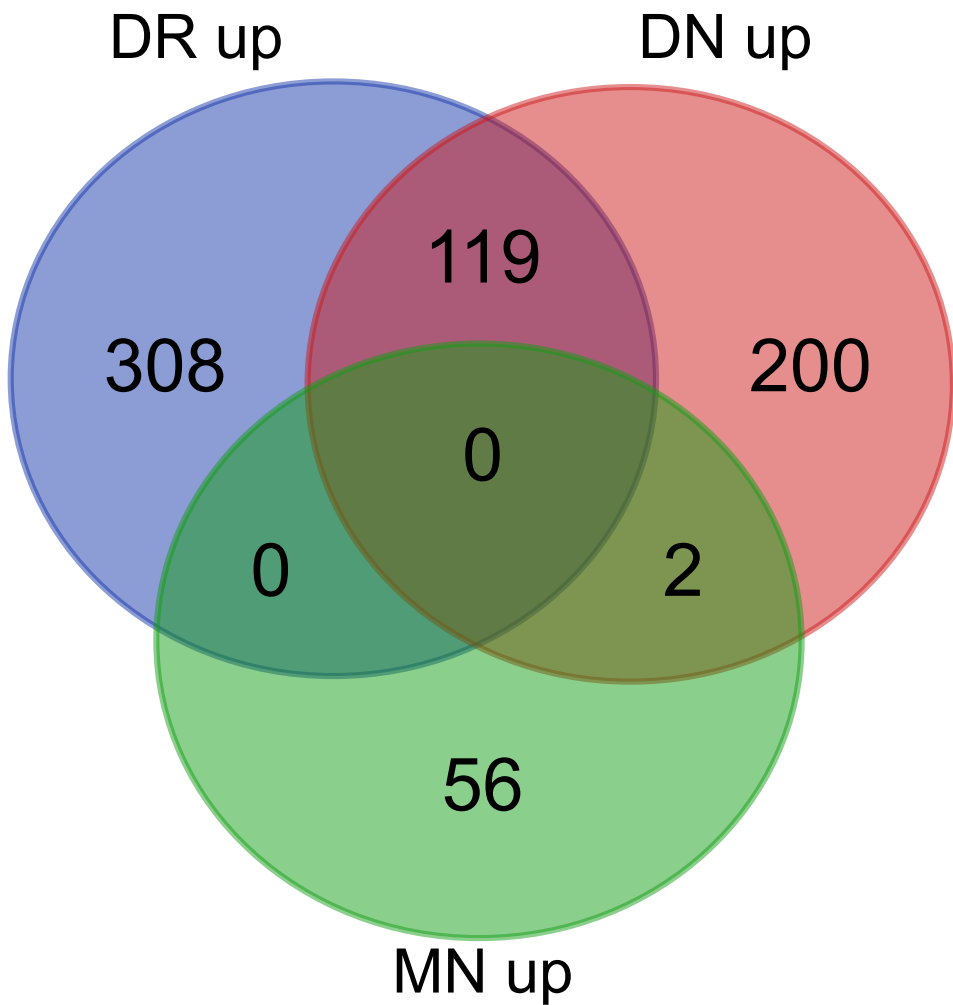

Supplement: Supplementary file 5 [file DataSheet3.pdf]

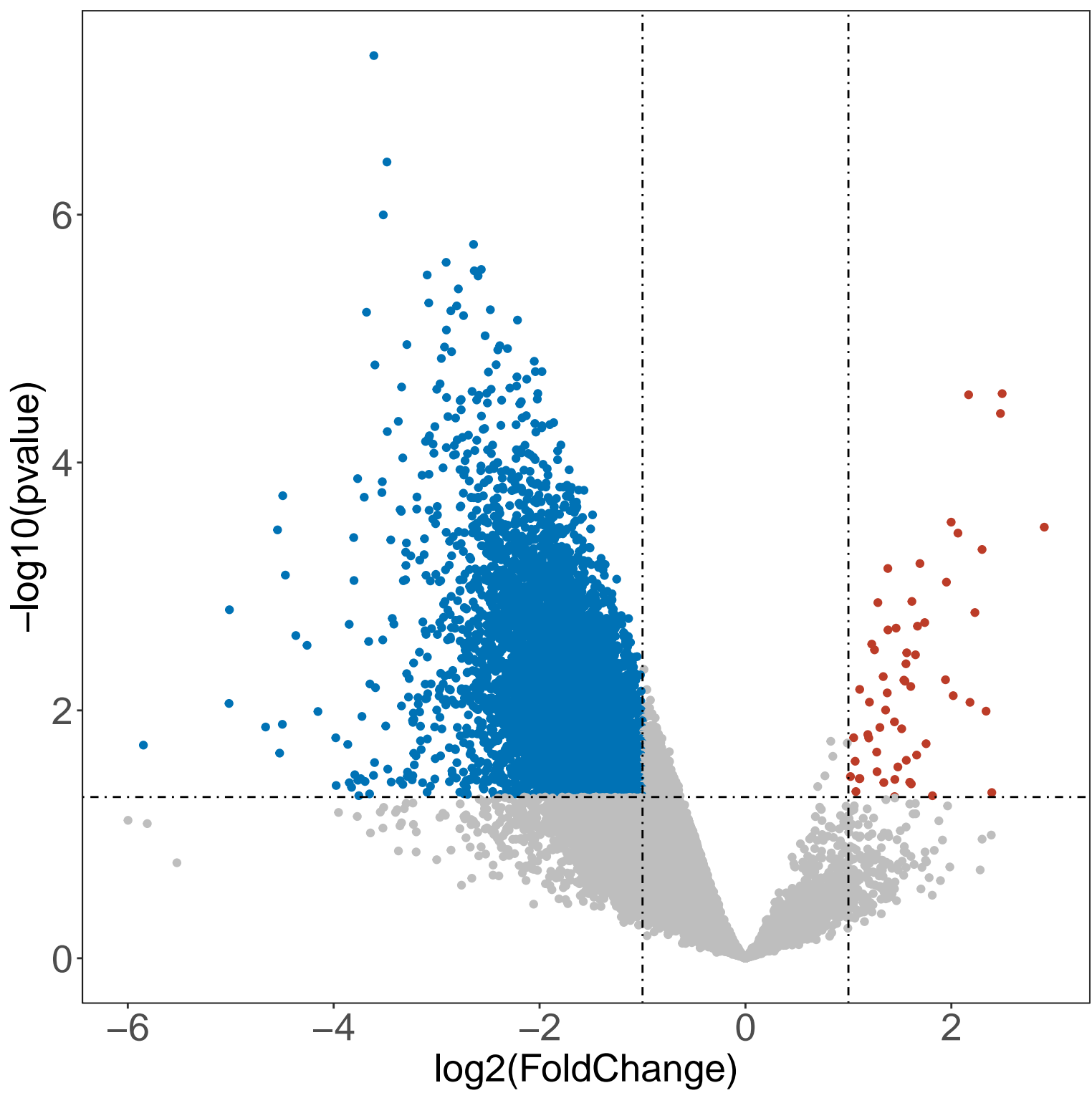

Supplement: Supplementary file 7 [file DataSheet1.pdf]

Type DN DR

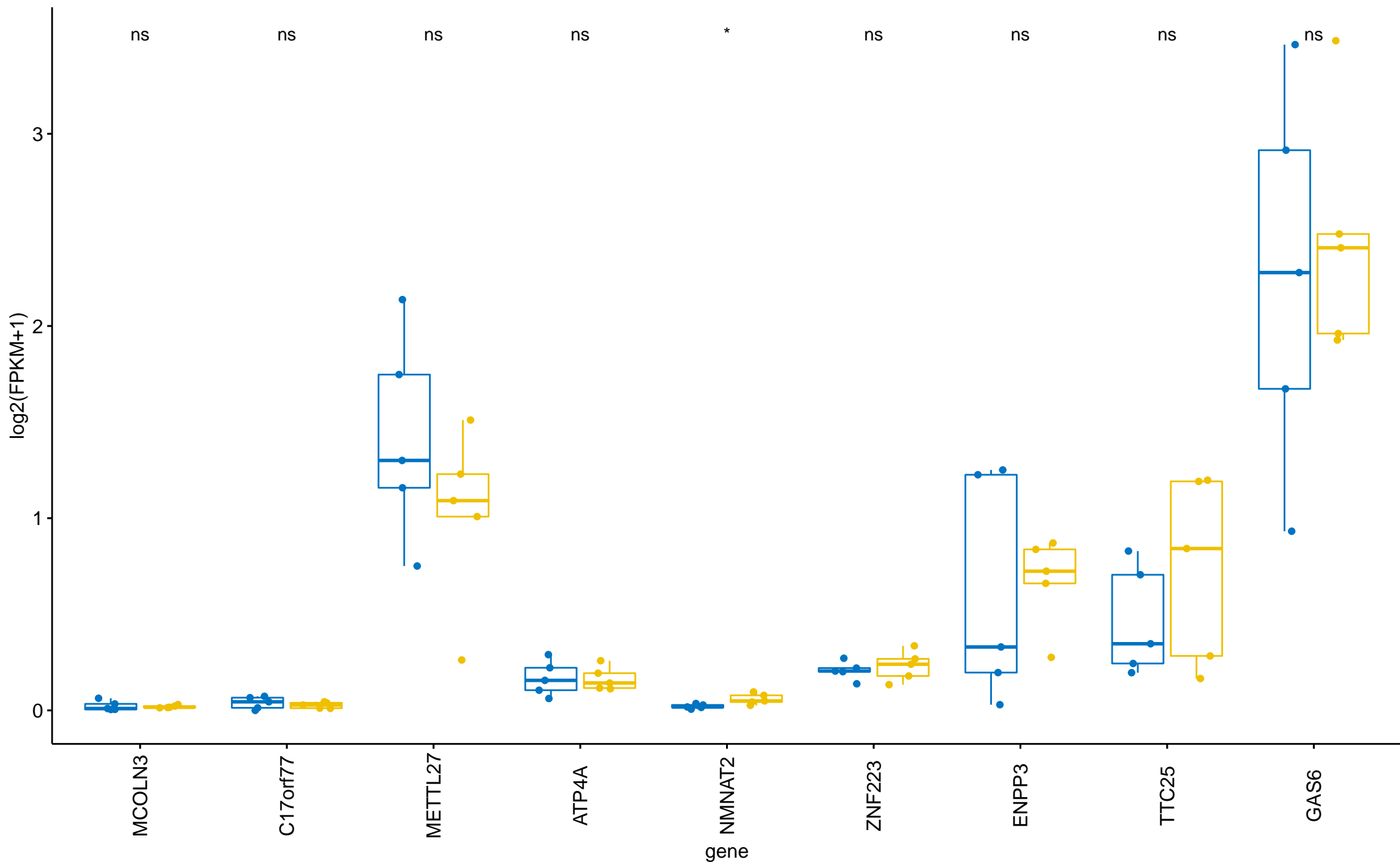

Supplement: Supplementary file 8 [file DataSheet5.pdf]
